# Associations between sleep and in-race gastrointestinal symptoms: an observational study of running and triathlon race competitors

**DOI:** 10.5935/1984-0063.20200029

**Published:** 2020

**Authors:** Patrick Benjamin Wilson

**Affiliations:** Old Dominion University, Human Movement Sciences - Norfolk - VA - United States.

**Keywords:** Anxiety, Exercise, Pain, Sleep, Stomach

## Abstract

**Objective:**

It remains unstudied whether poor sleep is involved in the etiology of gastrointestinal (GI) problems in athletes.

**Methods:**

Eighty-seven running and triathlon/duathlon race (>60 minutes) participants completed questionnaires to quantify the Sleep Problems Index-(SPI)-I and sleep parameters from the night before races. For GI symptoms, participants reported the severity (0-10 scale) of four upper and three lower symptoms during races. Spearman’s correlations examined whether sleep measures were associated with in-race GI symptoms. Partial correlations were calculated to control for age, resting GI symptoms, and anxiety.

**Results:**

SPI-I scores correlated with in-race upper GI symptoms (rho=0.26, p=0.013). Controlling for anxiety attenuated this association (rho=0.17, p=0.117), while other control variables had little effect. Acute sleep quantity and quality were not associated with GI symptoms.

**Conclusions:**

Chronic sleep dysfunction is modestly correlated with in-race upper GI symptoms, though future research should clarify whether this is mediated or moderated by factors like anxiety

## INTRODUCTION

Adequate sleep is recognized to be important to athlete health and performance. Acute sleep loss, for example, results in reductions in many aspects of cognitive function^1^, and chronic sleep restriction is associated with a higher risk of injury in athletes^1^. Despite the benefits of achieving adequate sleep (e.g., 7-8h/night), some athletes will inevitably underestimate the detrimental consequences of failing to consistently obtain recommended amounts of sleep.

Although several detrimental impacts of inadequate sleep quality and quantity on athlete health and performance are documented in the scientific literature, surprisingly little research has evaluated how these factors impact gastrointestinal (GI) function in this population. The occurrence of GI symptoms during exercise and competition varies depending on a host of factors such as age, anxiety/stress, environmental conditions, exercise intensity, and pre-exercise and during-exercise nutritional intake^2-4^. Irrespective of cause, most athletes (particularly those partaking in prolonged running) report dealing with GI symptoms like nausea, reflux, bloating, intestinal cramping, gas and urges to defecate at some point during training or competition^5^.

Whether sleep disturbances play a role in the development of these GI problems is yet to be determined, but there is ample reason to suspect that associations would exist between sleep dysfunction and GI disturbances in athletes. For example, chronic sleep dysfunction is associated with a higher incidence and/or severity of GI symptoms among the general population^6^, and similar results have been found in people suffering from irritable bowel syndrome (IBS)^7^. The mechanisms responsible for these links remain understudied, but some investigations have found alterations in anorectal motility and heightened visceral sensitivity to physical and chemical stimuli among people experiencing sleep disruption^8^. These increases in visceral sensitivity could be explained, in part, by the effects of sleep loss on the activity of the endogenous opioid system^9^. Another possible explanation comes via the visceral theory of sleep, which posits that the central nervous system switches from primarily analyzing exteroceptive information during wakefulness to analyzing interoceptive information from the gut and other visceral organs during sleep^10^; thus, disruptions in sleep could interfere with interoceptive processing, which is believed to play a role in regulating GI function. Regardless of the specific mechanisms, sleep deprivation has been shown to consistently worsen perceptions of general pain and discomfort^11^, a finding that is also likely to apply to certain GI symptoms like heartburn, intestinal cramping and bloating.

Given the lack of research on the links between sleep and GI function in athletes, this preliminary observational investigation aimed to examine whether acute sleep duration and quality, as well as chronic sleep dysfunction, were associated with GI symptoms during prolonged (>60 minute) running and triathlon/duathlon races. It was hypothesized that poor acute sleep (quality and quantity) and chronic sleep dysfunction would be associated with a greater severity of GI symptoms during races, even after adjustment for several factors (anxiety, age, resting GI symptoms) known to impact GI symptomology in athletes.

## MATERIAL AND METHODS

### General Design and Participants

This study employed an observational, retrospective survey-based design. A web-based survey was designed and distributed through Qualtrics Software (Qualtrics, Provo, UT, USA). Participants were recruited through communications with directors of races and running clubs. Race directors/personnel and club organizers forwarded the survey hyperlink to racers or posted the hyperlink on social media.

To be eligible, individuals had to be aged 18 years or older and must have recently completed an exercise (e.g., running, cycling, triathlon/duathlon) race lasting at least one hour. There was no upper age limit exclusion criterium, but no athletes older than 70-years-old volunteered. In addition, participants had to complete the survey on the day of their race, sometime between when they finished the race and 11:59pm. that same evening. The completion-timing requirement was needed to ensure that questions about acute sleep duration and quality were answered in relation to the night before the races.

The protocol for this study was submitted to the Human Subjects Review Committee of the Darden College of Education and Professional Studies at Old Dominion University and was determined to be exempt from full IRB review according to federal regulations. Descriptive information for the 87 participants who completed all the necessary aspects of the survey is shown in Table 1.

**Table 1:** Demographic, competition, anxiety, and sleep-related variables for the participants.

	Men (n = 31)	Women (n = 56)
Age (years)	43.0 ± 11.6	37.6 ± 9.3
Race (%)		
Non-Hispanic white	25 (81%)	51 (91%)
Mexican American or Other Hispanic	4 (13%)	2 (4%)
Asian	1 (3%)	1 (2%)
Other	1 (3%)	2 (4%)
BMI (kg/m^2^)	23.9 ± 2.8	23.6 ± 3.5
Experience running (years)	12.4 ± 7.8	8.5 ± 7.7
Type of race (%)		
Run	23 (74%)	39 (70%)
Duathlon or triathlon	8 (26%)	17 (30%)
Race finish time (min)	140 ± 67	172 ± 74
STICSA-Trait (21-84)	31.7 ± 7.1	34.0 ± 8.6
SCAT (10-30)	18.6 ± 4.5	20.1 ± 5.8
SPI-I (0-100)	31.7 ± 10.9	29.5 ± 14.8
Prior night sleep duration (min)	380 ± 83	417 ± 97
Prior night sleep quality (1 = very badly, 6 = very well)	4.2 ± 1.2	4.2 ± 1.1

BMI: Body mass index; SCAT: Sport Competition Anxiety Test; SPI-I: Sleep Problems Index 1; STICSA-Trait: State-Trait Inventory for Cognitive and Somatic Anxiety. Mean ± standard deviation.

### Survey Contents

The survey began by inquiring about demographics such as age, gender and race/ethnicity. Participants then reported their height, weight, years of experience competing in similar competitions and the presence of any medical condition(s) causing them frequent GI symptoms.

Individuals were not excluded from the study based on these types of medical conditions (e.g., irritable bowel syndrome, dyspepsia, celiac disease, etc.), because as many as 30 million Americans or more are diagnosed with these conditions^12^. Next, respondents reported the type and distance of race they participated in, their finishing time, and whether they took any non-steroidal anti-inflammatory (NSAID) drugs on the morning of the race or during the race itself.

Questions about GI symptoms were based on a previous study from Wilson^5^. A 0-to-10 scale with descriptors of ‘no discomfort’ at 0, ‘moderate discomfort’ at 5, and ‘unbearable discomfort’ at 10 was used when eliciting information about the occurrence of seven different symptoms: nausea, regurgitation/reflux, stomach fullness, bloating, abdominal cramps, gas/flatulence and urge to defecate. The following standardized definitions were provided to the participants.

- Nausea: a feeling of sickness in the stomach marked by an urge to vomit.
- Regurgitation/Reflux: sensation of food or fluid returning from the stomach to the esophagus or mouth.
- Stomach fullness: a sensation of fullness or abdominal pressure in the upper abdomen.
- Bloating: a feeling of distension from a buildup of gas in the gut.
- Abdominal cramps: pain or cramping sensation, often experienced in the mid or lower portion of the abdomen.
- Gas/Flatulence: gas or flatus expelled through the anus.
- Urge to Defecate: sensation of needing to pass a bowel movement.

Participants first reported the severity of GI symptoms experienced during the race they just completed, followed by the severity of symptoms at rest over the past month. The questions about GI symptoms were followed by two standardized questionnaires related to general and competition anxiety. Anxiety has been reported to impact both GI symptom development^2,3^ and sleep, so it represented an important covariate to measure. The trait version of the State-Trait Inventory for Cognitive and Somatic Anxiety (STICSA) was used to assess general anxiety^13^, while the Sport Competition Anxiety Test (SCAT) was used to assess competition-related anxiety^14^.

Sleep assessments were made using questions based on the Saint Mary’s Hospital (SMH) Sleep Questionnaire^15^ and the Medical Outcomes Study (MOS) Sleep Scale^16^. Acute quantity of sleep was calculated from times that participants reported falling asleep and waking prior to races. The quality of sleep from the night before races was rated on a 6-point Likert scale (1 = very badly; 2 = badly; 3 = fairly badly; 4 = fairly well; 5 = well; 6 = very well) from the SMH Sleep Questionnaire. Chronic sleep dysfunction was quantified via the Sleep Problems Index-(SPI)-I, which is based on six items from the MOS Sleep Questionnaire. These six items measure the following issues: getting enough sleep to feel rested upon awakening; trouble falling asleep; difficulty falling back asleep after awakening prematurely; trouble staying awake during the day; awakening short of breath or with a headache; and getting enough sleep to function. The period covered by these items is four weeks, and respondents were asked to report how often each item applied to them (ranging from ‘none of the time’ to ‘all of the time’). Reponses to the items were converted to a 0-100 scale, and the items were averaged to generate SPI-I scores.

### Statistical Analysis

The normality of the data was determined by visually inspecting histograms and examining results from the Shapiro-Wilk test. Because the GI symptom data showed a right-skewed distribution with zero values, a non-parametric approach was used to examine the associations between sleep variables and GI-symptom data. Specifically, Spearman’s rank-order correlations were run between several sleep-related variables (previous night’s sleep duration, previous night’s sleep quality, SPI-I scores) and GI symptom ratings during the races. To reduce the total number of statistical analyses that were carried out, ratings of individual GI symptoms were combined into upper and lower GI categories. Nausea, regurgitation/reflux, stomach fullness and bloating were considered upper GI symptoms, while abdominal cramps, gas/flatulence and urge to defecate were considered lower GI symptoms. Possible scores for these categories were 0-40 for upper GI symptoms and 0-30 for lower GI symptoms.

In addition, to these uncontrolled bivariate correlations, partial rank-order correlations were calculated to examine how the magnitude of any significant associations between sleep variables and GI symptoms changed after controlling for potential confounders. To identify appropriate control variables, regular Spearman’s rank-order correlations were run between potential confounders (of continuous or ordinal nature) and in-race GI symptoms. All continuous or ordinal variables that were significantly correlated with in-race GI symptoms were subsequently included in the partial rank-order correlation analyses. These control variables included age, resting GI symptoms, STICSA scores and SCAT scores. The dichotomous variables gender, type of race (run vs. triathlon/duathlon), and NSAID use (yes vs. no) were not included as control variables because in-race GI symptoms did not significantly differ by these factors (based on Mann-Whitney U tests). Although resting GI symptoms were different between participants with and without GI-related medical diagnoses (the specific data are reported in the results section), the presence of these disorders (yes vs. no) was not included as a control variable because resting GI symptoms were already included in the partial correlation analyses.

The analyses were completed using SPSS version 26 (IBM, Armonk, NY, USA). A *p*-value ≤ 0.05 was used to determine statistical significance.

## RESULTS

As shown in Table 1, the sample was 64% female and of predominantly white race. Over 70% of respondents participated in a running race, while roughly 29% completed a triathlon or duathlon. Only seven participants (8%) reported sleeping less than 5h on the night prior to their races, and mean scores for subjective sleep quality (4.2 out of 6) correspond to ‘fairly well’ to ‘well’ ratings. Twelve (14%) participants reported having some sort of medical condition that caused them GI symptoms; 4 had IBS, 2 had lactose intolerance, 1 had Crohn’s disease, 1 had endometriosis, 1 had Celiac disease, 1 had reflux disease, 1 had constipation and 1 had generalized anxiety disorder. Based on Mann-Whitney U tests, participants with these GI-related medical diagnoses reported higher upper (median: 8.5 vs. 2 out of 40; Z-statistic -3.43, *p*=0.001) and lower (median: 11 vs. 2 out of 30; Z-statistic -3.12, *p*=0.002) GI symptoms at rest. Eighteen participants (21%) used NSAIDs on the morning of their races and/or during races.

Table 2 shows the extent to which potential GI symptom predictors correlated with cumulative upper and lower GI symptom ratings during races. For upper GI symptoms, six of the variables were significantly correlated with symptom scores, with STICSA scores showing the strongest correlation (rho=0.33; *p*=0.002). Of the three sleep-related variables, only scores on the SPI-I were significantly correlated with upper GI symptoms (rho=0.26, *p*=0.013). With respect to lower GI symptoms, four variables were correlated with symptom scores, and resting lower GI symptom ratings showed the strongest association with in-race lower GI symptoms (rho=0.36, *p*=0.001).

**Table 2:** Correlations between in-race GI symptoms and various predictor variables.

	In-race upper GI symptoms	In-race lower GI symptoms
Age	-0.23*	-0.23*
BMI	-0.04	0.13
Experience	-0.06	-0.15
Race finish time	-0.08	-0.01
STICSA-Trait	0.33*	0.23*
SCAT	0.28*	0.22*
Resting upper GI symptoms	0.24*	0.21
Resting lower GI symptoms	0.25*	0.36*
Duration of previous night's sleep	0.01	-0.12
Sleep quality	-0.09	-0.01
SPI-I	0.26*	0.13

BMI: Body mass index; SCAT: Sport Competition Anxiety Test; SPI-I: Sleep Problems Index 1; STICSA: Trait version of the State-Trait Inventory for Cognitive and Somatic Anxiety. Sleep quality ranged from 1 (very badly) to 6 (very well).

* *p*<0.05.

The partial rank-order analysis revealed that scores on the SPI-I were no longer significantly correlated with in-race upper GI symptoms after accounting for age, STICSA scores, SCAT scores, and resting upper GI symptom ratings (rho=0.17, *p*=0.124). Notably, this attenuation of the correlation was driven almost entirely by the inclusion of STICSA scores.

This is supported by the fact that a partial rank-order correlation that included STICSA as the sole control variable showed a similar attenuation (rho=0.17, *p*=0.117) as compared to the partial correlation that included all four control variables. In addition, partial rank-order correlations between in-race upper GI symptoms and SPI-I scores that included SCAT scores (rho=0.24, *p*=0.027), age (rho=0.27, *p*=0.012), and resting upper GI symptoms (rho=0.24, *p*=0.027) as sole control variables all remained statistically significant and largely unattenuated when compared to the fully adjusted partial correlation.

## DISCUSSION

This is the first investigation, to the knowledge of the author, to show that chronic sleep problems are associated with GI symptoms experienced during sporting competition, in this case prolonged running and triathlon/duathlon racing. Several considerations should be taken into account when interpreting these findings. First, the correlation between the SPI-I and in-race GI problems was significant for upper GI but not lower GI symptoms. Second, the correlation between the SPI-I and in-race upper GI symptoms was modest in size (rho=0.26) and became insignificant after adjusting for trait anxiety. Still, these findings agree with research in the general population that has found associations between chronic sleep problems and GI disturbances. Cremonini et al.^6^, for example, found that sleep scores based on questions from the Insomnia Severity Index were positively correlated with overall GI symptom scores, and the size of the correlation (partial *r* = 0.28) was very similar to the correlation in this investigation.

Although the correlation between chronic sleep problems and in-race GI symptoms was modest, it is important to keep in mind that most other documented predictors of GI symptoms during competition and training are also modest in size. For example, a study of 53 people participating in a 70.3-mile triathlon found that intakes of dietary energy, carbohydrate, and caffeine were modestly correlated (rho=0.28-0.36) with GI symptoms experienced during the run and bicycle portions of the race^17^. Likewise, Wilson^2^ found modest correlations between the frequency of substantial GI distress during runs over a 30-day period and several factors, including age (rho=-0.30), years of running experience (rho=-0.17), average run intensity (rho=0.23), perceived stress ratings (rho=0.29), and anxiety ratings (rho=0.27). Perhaps the best explanation for these observations is that each GI symptom has multiple potential causes, and any single variable is likely to explain only a relatively small proportion of the observed variance in GI symptomology.

An interesting secondary finding of this study is that adjustment for anxiety scores, as measured by the STICSA, significantly attenuated the association between chronic sleep problems and upper GI symptoms during races. After including STICSA scores in a partial correlation analysis, the size of the rho decreased to 0.17, which contrasts against a rho of 0.26 in the unadjusted analysis. This attenuation of the association between sleep problems and GI symptoms after adjusting for anxiety has also been found in non-athletes^18^. Given the documented interrelationships between these three factors (sleep, GI symptoms, anxiety), as well as the possibility that these variables could influence each other in a bidirectional manner, it is challenging to determine the extent to which they are causally related. That said, a single night of sleep restriction in patients with gastroesophageal reflux disease^19^ as well as 12 consecutive days’ worth of sleep restriction in healthy adults have been shown to increase GI-related pain and discomfort^20^. These findings provide circumstantial evidence to support the notion that the chronic sleep dysfunction experienced by some of the participants in this study directly exacerbated in-race upper GI symptoms. However, this hypothesis needs to be verified by additional observational studies and perhaps through controlled trials, although experiments in humans that induce chronic sleep restriction pose substantial practical challenges.

An additional finding of this study is that neither acute sleep duration nor acute sleep quality was related to the severity of in-race upper or lower GI symptoms. There are several potential reasons for this observation. First, only a small percentage of this study’s participants experienced acute sleep deprivation (e.g., less than five hours of sleep). Second, this study’s measures of sleep duration and quality were based on self-report, which could have contributed to inaccuracies for some participants. Of these potential explanations, it seems more plausible that the first - most participants were not sleep-deprived - explains the results of this study. Alternatively, it could be that multiple days of poor sleep are needed to elicit a worsening of GI symptoms.

Given the observational nature of this work, it is impossible to determine whether the associations between sleep dysfunction and in-race GI symptoms are causal. Although a few important confounding variables were accounted for in the analyses, several others were not, perhaps the most important being pre-race and in-race nutritional intakes. Requiring detailed assessments of nutritional intakes would have significantly added to survey completion time.

Furthermore, the sleep assessments were based on subjective self-report measures. Given the difficulty of objectively assessing sleep quality and quantity over several weeks, subjective measures of sleep are the main methodology used in most studies on chronic sleep issues.

Another limitation is that the GI symptoms were reported retrospectively as opposed to truly being collected in-race. That said, it has been shown that the GI symptom scale used in this study is valid, even when used in a retrospective fashion^5^. Finally, this study’ statistical approach was based on non-parametric correlational analyses and did not examine mediation or moderation effects. These more complicated analyses were deemed unsuitable considering the modest sample size of the study and the skewed, transformation-resistant nature of the GI symptom data.

## CONCLUSIONS

In addition to the more well-documented negative consequences of chronic sleep dysfunction in athletes, it appears as though upper GI symptoms experienced by race competitors may, to a modest extent, result from chronic sleep problems. These findings have implications for athletes who experience disturbed sleep over a period of several days or weeks due to travel, stress and other factors known to impact sleep. Yet, these preliminary observations need to be confirmed in other samples of athletes. Furthermore, future studies should attempt to clarify the extent to which anxiety mediates the relationship between sleep dysfunction and GI dysfunction in athletes.
